# Illustrating the biological functions and diagnostic value of transmembrane protein family members in glioma

**DOI:** 10.3389/fonc.2023.1145676

**Published:** 2023-03-31

**Authors:** Ying Zhang, Wei Zhang, Qiyou Yuan, Wenqing Hong, Ping Yin, Tingting Shen, Lutong Fang, Junlan Jiang, Fangxiao Shi, Weiwei Chen

**Affiliations:** ^1^ Department of Pathology, First Affiliated Hospital of Anhui Medical University, Hefei, Anhui, China; ^2^ Department of Neurology, First Affiliated Hospital of Anhui Medical University, Hefei, Anhui, China; ^3^ Department of Neurosurgery, First Affiliated Hospital of Anhui Medical University, Hefei, Anhui, China; ^4^ Department of Health Management Center, First Affiliated Hospital of Anhui Medical University, Hefei, Anhui, China; ^5^ School of Materials & Science, Beijing Institute of Technology, Beijing, China

**Keywords:** Glioma, transmembrane protein, prognostic signature, immunity, TMEM158

## Abstract

**Background:**

It is well-established that patients with glioma have a poor prognosis. Although the past few decades have witnessed unprecedented medical advances, the 5-year survival remains dismally low.

**Objective:**

This study aims to investigate the role of transmembrane protein-related genes in the development and prognosis of glioma and provide new insights into the pathogenesis of the disease

**Methods:**

The datasets of glioma patients, including RNA sequencing data and relative clinical information, were obtained from The Cancer Genome Atlas (TCGA), Chinese Glioma Genome Atlas (CGGA) and Gene Expression Omnibus (GEO) databases. Prognostic transmembrane protein-related genes were identified by univariate Cox analysis. New disease subtypes were recognized based on the consensus clustering method, and their biological uniqueness was verified *via* various algorithms. The prognosis signature was constructed using the LASSO-Cox regression model, and its predictive power was validated in external datasets by receiver operating characteristic (ROC) curve analysis. An independent prognostic analysis was conducted to evaluate whether the signature could be considered a prognostic factor independent of other variables. A nomogram was constructed in conjunction with traditional clinical variables. The concordance index (C-index) and Decision Curve Analysis (DCA) were used to assess the net clinical benefit of the signature over traditional clinical variables. Seven different softwares were used to compare the differences in immune infiltration between the high- and low-risk groups to explore potential mechanisms of glioma development and prognosis. Hub genes were found using the random forest method, and their expression was based on multiple single-cell datasets.

**Results:**

Four molecular subtypes were identified, among which the C1 group had the worst prognosis. Principal Component Analysis (PCA) results and heatmaps indicated that prognosis-related transmembrane protein genes exhibited differential expression in all four groups. Besides, the microenvironment of the four groups exhibited significant heterogeneity. The 6 gene-based signatures could predict the 1-, 2-, and 3-year overall survival (OS) of glioma patients. The signature could be used as an independent prognosis factor of glioma OS and was superior to traditional clinical variables. More immune cells were infiltrated in the high-risk group, suggesting immune escape. According to our signature, many genes were associated with the content of immune cells, which revealed that transmembrane protein-related genes might influence the development and prognosis of glioma by regulating the immune microenvironment. TMEM158 was identified as the most important gene using the random forest method. The single-cell datasets consistently showed that TMEM158 was expressed in multiple malignant cells.

**Conclusion:**

The expression of transmembrane protein-related genes is closely related to the immune status and prognosis of glioma patients by regulating tumor progression in various ways. The interaction between transmembrane protein-related genes and immunity during glioma development lays the groundwork for future studies on the molecular mechanism and targeted therapy of glioma.

## Introduction

Glioma accounts for the largest proportion of malignant craniocerebral tumors (1), with high invasiveness and lethality (2). Clinical cancer research has made great progress in recent years. Nonetheless, the overall survival (OS) remains poor (3). Current treatment alternatives include radical surgical resection, radiotherapy, and chemotherapy. However, these conventional approaches exhibit many limitations in treating this malignant disease, with high recurrence risks. Moreover, radiotherapy and chemotherapy can lead to the toxicity of other important organs, resulting in the lowered life quality of patients. Therefore, emphasis should be placed on comprehensive and individualized treatment, which emphasizes the importance of the quest to identify more specific biomarkers for glioma.

The transmembrane protein (TMEM) family refers to a group of proteins that span the lipid bilayer; however, their structure, biological functions and effects have been largely underinvestigated (4). TMEMs have been documented in all kinds of cells, cytomembranes, the mitochondria, endoplasmic reticulum (ER), and lysosome or Golgi apparatus. Some studies have revealed that different TMEM genes are up or downregulated in cancers, suggesting their potential as tumor suppressors or promoters. Their role has also been described as chemotherapeutic resistance and response to anticancer treatments (5). Current evidence suggests that TMEM proteins are involved in cancer-related signaling pathways, significantly impacting cancer metastasis, recurrence, and patient survival. In ovarian cancer, TMEM119 has been demonstrated to promote tumor cell proliferation, invasion and migration by activating the PDGFRB/PI3K/AKT signaling pathway (6). In hepatocellular carcinoma, TMEM206 upregulation has been associated with poorer patient prognosis (7). What’s more, TMEM43 upregulation has been closely associated with malignant brain tumors, and the inhibition of TMEM43 expression inhibited the growth of brain tumor cells *in vitro* and *in vivo* (8). Hence, identifying TMEMs involved in tumor development and progression is a promising approach to finding new targets for glioma therapy.

In this study, we probed different public online datasets from the GEO, TCGA and CGGA databases. 22 TMEM genes were identified in glioma patients, and a prognostic signature was established based on six prognosis-related TMEM genes. The relationship between the signature and clinical attributes and cancer characteristics was assessed. Our signature could accurately predict the clinical outcomes of glioma patients and could be used as an independent prognostic factor, providing new insights for exploring the molecular mechanisms and targeted treatments in this patient population. The random forest method showed that TMEM158 is a potential biomarker for diagnosing and treating glioma.

## Materials and methods

### Data retrieval and preprocessing

First, HTSeq-FPKM gene expression data and relative clinical information were downloaded from The Cancer Genome Atlas (TCGA) database (https://portal.gdc.cancer.gov/) as the training group. After patients without complete survival data were excluded, 631 glioma patients with complete follow-up data and follow-up time longer than 30 days were enrolled. During the validation process, the same inclusion criteria were adopted, from the Chinese Glioma Genome Atlas (CGGA) database (http://www.cgga.org.cn/), 618 glioma patients were included from the dataset CGGA-693 and 306 glioma patients from the dataset CGGA-325. 249 glioma patients were enrolled in the dataset GSE16011 (https://www.ncbi.nlm.nih.gov/geo/query/acc.cgi?acc=GSE16011) downloaded from the Gene Expression Omnibus (GEO) database. Using the “Combat” algorithm from the R package “sva”, batch effects due to the abiotic bias were removed among the TCGA-glioma, CGGA-693, CGGA-325, and GSE16011 datasets (9). Finally, 22 transmembrane protein-related genes were obtained and 14 present in all four datasets (ANO1, TMEM17, TMEM25, TMEM45A, TMEM88, TMEM97, TMEM98, TMEM140, TMEM156, TMEM158, TMEM176A, TMEM43, TMEM116) (10).

### Identification of transmembrane proteins-related subtypes to validate the value of transmembrane proteins-related genes in glioma

The R package “survival” was used to conduct the univariate Cox and Kaplan Meier (KM) survival analyses in the TCGA-glioma, CGGA-693, CGGA-325, and GSE16011 datasets. To ensure the accuracy of the results, a P value< 0.001 was used as the screening criteria to identify transmembrane protein-related prognostic genes. The R package “ConsensusClusterPlus” was used for unsupervised clustering to identify new subtypes to further understand the prognostic value of transmembrane protein-related prognostic genes (11). The number of new subgroupswase was based on the consensus cumulative distribution function (CDF) plot, delta area plot, and cluster consensus. The packages “survival” and “survminer” were applied to conduct KM survival analysis and the log-rank test on transmembrane protein subtypes. The heatmap showed the differential expression profiles of all transmembrane protein-related prognostic genes in the four subtypes. Principal Component Analysis (PCA) was performed to reduce the dimensionality of the data and verify differential expression patterns among various subtypes. The Microenvironment Cell Populations-counter (MCP-Counter) method was used to evaluate the abundance of immune cells in the subtypes (12). Moreover, the Estimation of Stromal and Immune cells in Malignant Tumors using Expression data (ESTIMATE) algorithm was adopted to estimate mesenchymal and immune cells in malignant tumor tissues and calculate the tumor purity of different molecular subpopulations (13). In addition, we applied the gene set variation analysis (GSVA) algorithm to compare the tumor microenvironment (TME) differences (14, 15). Moreover, we compared the differential HLA expression levels in different subtypes.

### Establishment, evaluation and implementation of the transmembrane protein-related gene signature

Using the TCGA-glioma dataset as the training cohort, the Lasso-Cox regression analysis was performed to screen out nine transmembrane proteins-related prognostic genes (TPRPGs), and the multicollinearity was eliminated. At last, a risk score signature was obtained by multiplying the β (Coef) value by the TPRPG expression value as follows (β1*TPRPG1+β2* TPRPG2 +β3* TPRPG3+⋯+βn* TPRPGn), where β refers to the coefficient of TPRPGs (16, 17). The median risk score was set as the threshold to divide 631 patients into high- or low-risk groups. Then KM survival and receiver operating characteristic (ROC) analyses were conducted. To determine whether the signature could be used as an independent prognosis index of clinical traits, including age, gender, and staging, univariate and multivariate Cox regression analyses were performed on age, gender, staging and the signature of glioma patients. Decision Curve Analysis (DCA) was conducted to assess whether the signature could benefit patients. Additionally, we used the R package “rms” to improve the accuracy during glioma prognosis prediction to construct a nomogram based on multivariate characteristics. The ROC and calibration curves were depicted to evaluate the accuracy of the nomogram.

### Exploration of the potential mechanisms of glioma

Seven different softwares (12, 18–23) were used to quantify and compare the abundance of immune infiltration between high- and low-risk groups. Moreover, the Pearson correlation between the signature genes and risk scores with immune cell contents was calculated. In a study by Thorsson V et al. (24), immunogenomics analysis was conducted on more than 10000 tumors, and six immune subtypes of pan-cancer (C1 (wound healing), C2 (IFN-g dominant), C3 (inflammatory), C4 (lymphocyte depleted), C5 (immunologically quiet), C6 (TGF-β dominant) were identified. These immune subtypes could be used to identify patterns of immune responses that may influence prognosis. Three main immune types were identified in the TCGA-glioma, namely C3 (inflammatory), C4 (lymphocyte depleted), and C5 (immunologically quiet). Finally, we assessed the distribution of each immune subtype in the at-risk population.

### Hub gene identification

According to the random forest algorithm, we sorted the genes in the signature based on the survival importance to determine hub genes (25). Moreover, the Tumor Immune Single-cell Hub (TISCH) database was used to locate the expression profiles of the hub gene at the single-cell level (26).

## Results

### The biological uniqueness of the four subtypes was verified based on the transmembrane protein transcription level

**Figure 1:**
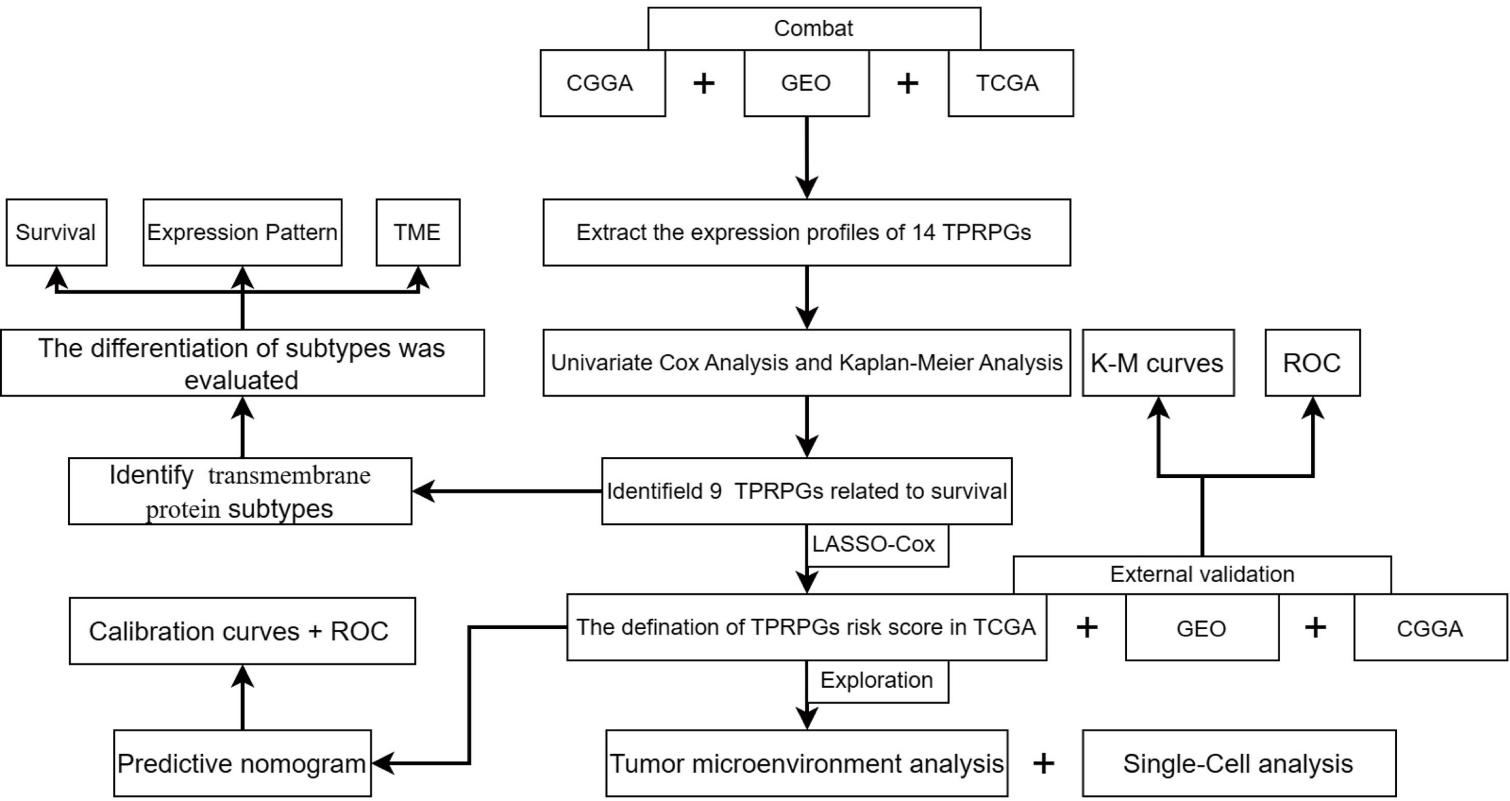
The work flowchart.

**Figure 2:**
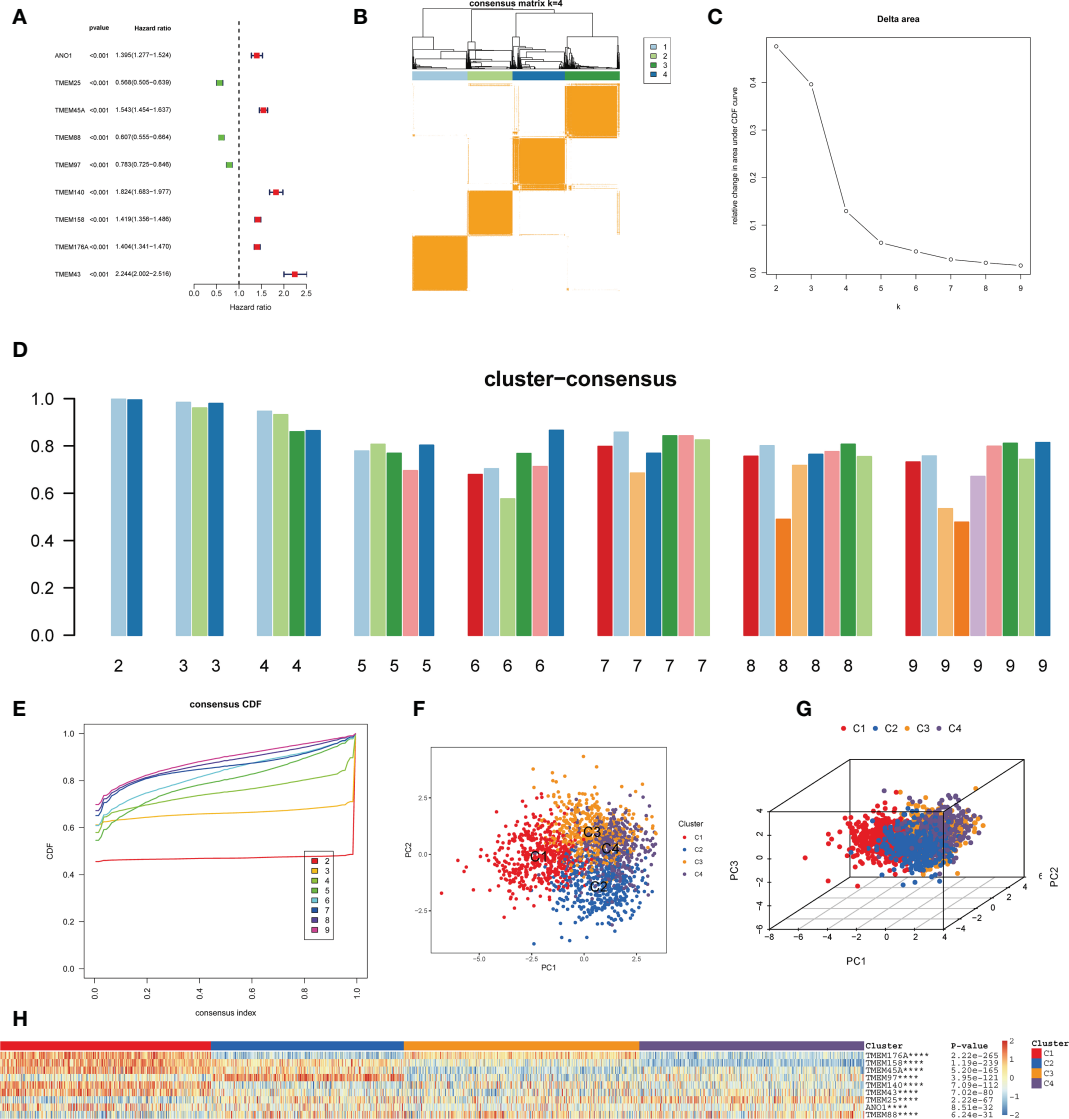
Identification of transmembrane protein-related subtypes. **(A)** Forest plot of nine prognostic transmembrane protein-related genes in glioma based on the univariate Cox regression analysis. **(B)** Subgroups of glioma defined by nine prognostic transmembrane protein-related genes. The 4 cohorts’ consensus score matrix for all samples when k =4. When two random samples had a higher consensus score in distinct interactions, they were more likely to be clustered. **(C)** The Delta Area Plot shows the relative change between k and k-1 relative to the area under the CDF curve. When k = 5, the area under the curve only increases slightly, so 4 was the appropriate k value. **(D)** Cluster-Consensus Plot shows the cluster-consensus value of each category under different k values (the mean of pairwise consensus values of members in the cluster). The higher (lower) the value represents, the higher (lower) the stability. It can determine the cluster-consensus value under the same k value and between different k values. k= 4 is the most suitable choice for satisfying both the maximum clustering and relatively high Cluster-Consensus conditions. **(E)** The consistency Cumulative Distribution Function (CDF) plot shows the cumulative distribution function when k takes different values, which is used to determine when k takes what value; the CDF reaches an approximate maximum when the clustering analysis results are the most reliable. Given the small k value for the CDF negative slope, k=4 was selected. **(F, G)** The principal component analysis (PCA) of glioma samples. The points of different colors represent samples of different groups. A closer distance between the points suggests that the expression of transmembrane protein-related genes is similar. **(H)** The heatmap of the expression of transmembrane protein-related genes in the four types of samples. The rows represent genes, the columns represent samples, red indicates high expression, blue indicates low expression, and the categories of samples are marked with different colors on the top of the heatmap. Survival analysis was performed using univariate Cox regression analysis. Molecular subtypes were identified using the unsupervised hierarchical clustering method. Differential analysis was conducted using the Kruskal-Wallis test. ****p < 0.0001.

**Figure 3:**
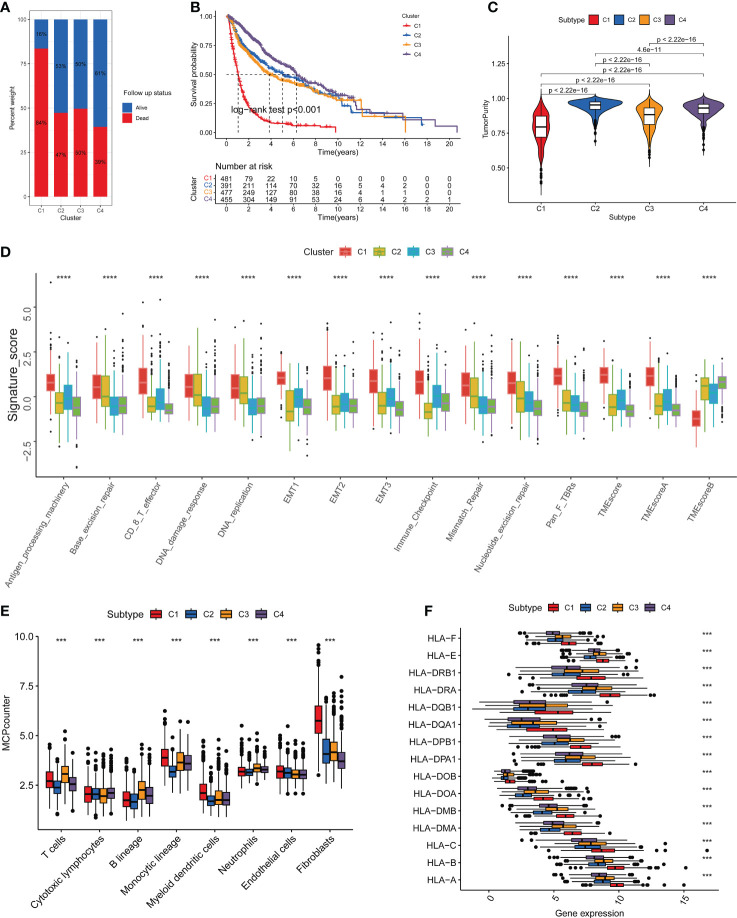
Discrimination of Different Subtypes. **(A)** The proportion of survival status of patients in four transmembrane protein-related subtypes. **(B)** Kaplan–Meier survival analysis of OS for patients with the four transmembrane protein-related subtypes. **(C)** Comparisons between the four subgroups in terms of tumor purity in tumor tissues. **(D)** Boxplots were used to display the expressions difference of tumor microenvironment signature. **(E)** Comparison of the abundance of immune-infiltrating cells among the four subgroups by the MCP-counter algorithm in tumor tissues. **(F)** Boxplots were used to display the expressions difference of HLA genes. ∗∗∗P < 0.001; ∗∗∗∗p < 0.0001. Survival analysis was conducted using the Kaplan-Meier method. **Figure 2C** adopted the Wilcoxon test method. Other Differential analyses were conducted using the Kruskal-Wallis test.

The flowchart of this study is shown in **Figure 1**. Nine key TPRPGs (ANO1, TMEM25, TMEM45A, TMEM88, TMEM97, TMEM140, TMEM158, TMEM176A, and TMEM43) were related to prognosis (**Figure 2A**). Based on the integrated results from the consensus cumulative distribution function (CDF) plot, delta area plot, and cluster-consensus, the optimal number of clusters was determined to be four, indicating the presence of four transmembrane protein-related subtypes (C1, C2, C3, C4)(**Figures 2B–E**). The PCA plots (**Figures 2F, G**) and the heatmap (**Figure 2H**) showed that the prognostic transmembrane protein-related genes exhibited differential expression in the four subgroups. Among all groups, most deaths were observed in C1 (**Figure 3A**). Based on KM survival analysis, C1 had the worst prognosis (**Figure 3B**). The tumor purity of C1 was the lowest, while it was the highest in C2 (**Figure 3C**). Consistent with the results from the ESTIMATE algorithm, almost all TME-related scores were highest in the C1 subgroup based on the signature (**Figure 3D**). The abundance of immune and non-immune cells was higher in C1 than in other subgroups (**Figure 3E**). Besides, HLA expression in C1 was the highest (**Figure 3F**).

### The transmembrane protein-associated signature is an independent risk factor for glioma

**Figure 4:**
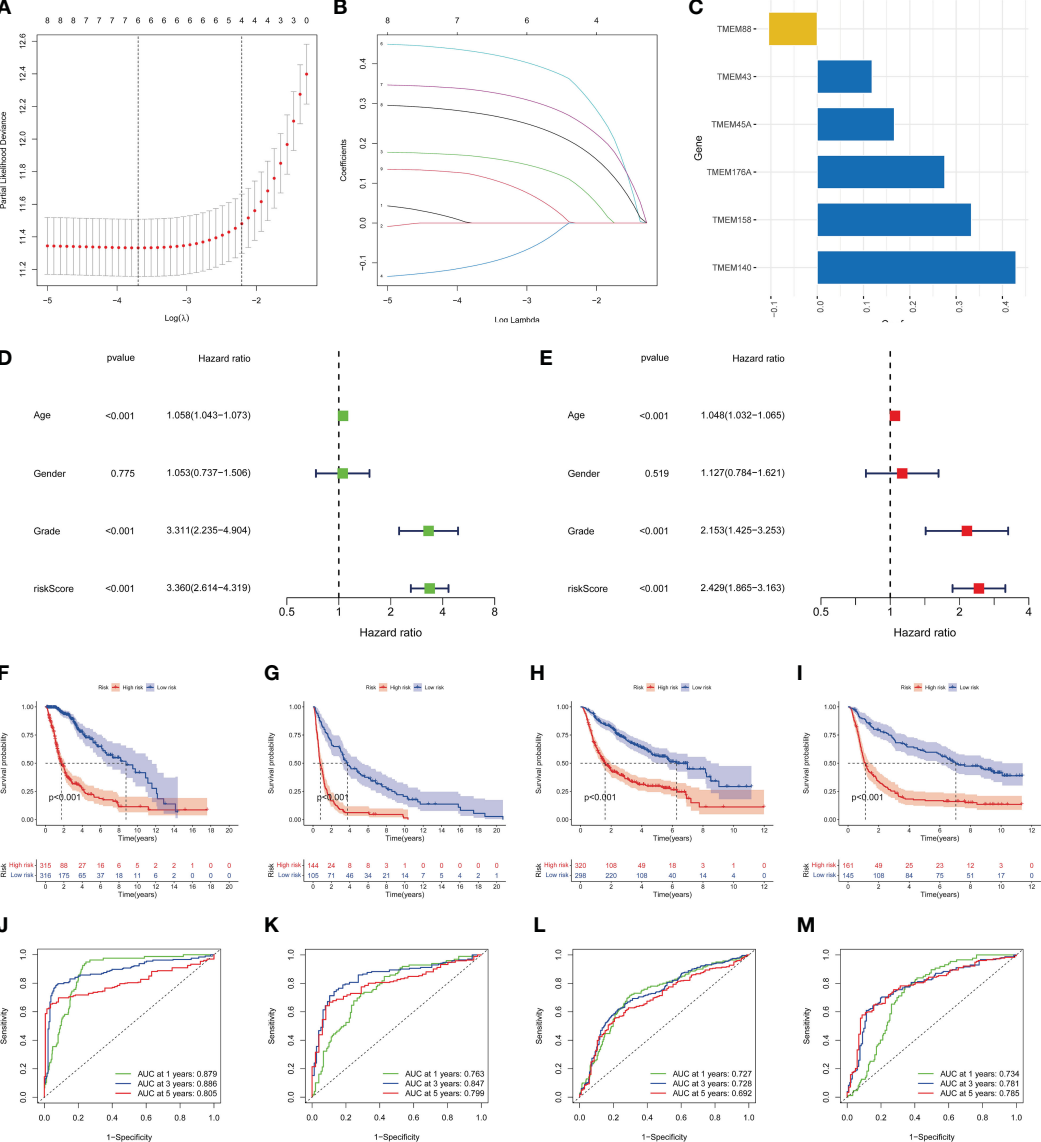
Identification of the transmembrane protein prognostic signature. **(A, B)** LASSO Cox regression analysis of the 9 OS-related transmembrane proteins. **(C)** The 6 genes included in the signature. Corresponding coefficients were depicted by horizontal bars, respectively. **(D, E)** Univariate and multivariate Cox regression revealed significant survival-related clinicopathological parameters in the forest plots diagram. **(F-I)** Kaplan–Meier OS curves with difference detection by log-rank test for patients from the training and validation datasets. TCGA-glioma, GSE16011, CGGA693, and CGGA325 datasets are arranged from left to right. **(J-M)** ROC analysis of the 6-genes signature in the four datasets.

The LASSO regression model was conducted using the expression profile data of 9 transmembrane protein-related prognostic genes, and the transmembrane protein-related prognostic risk signature was then established. The “cv.glmnet” function was used to perform 10-fold cross-validation to identify six genes (TMEM45A, TMEM88, TMEM140, TMEM158, TMEM176A, and TMEM43) with a regression coefficient. The risk scoring formula was as follows: risk score = (0.166782270191739*TMEM45A) + (-0.105110143608762*TMEM88) + (0.429175423075546*TMEM140) + (0.333132856396644*TMEM158) + (0.275424964997017*TMEM176A)+(0.118615470161412*TMEM43) (**Figures 4A–C**). Univariate (**Figure 4D**) and multivariate (**Figure 4E**) Cox regression analyses were used to distinguish independent risk factors. Results showed that age, staging and risk score were independent predictors of OS in glioma patients. All patients were divided into high- or low-risk groups according to the median value in the training group. KM curves showed that the low-risk group had a relatively better prognosis (**Figures 4F–I**). Moreover, the 1-, 2-, and 3-year area under curve (AUC) values of the 6-gene risk signature yielded satisfactory sensitivity and specificity in both the training group and the external validation set (**Figures 4J–M**).

### The transmembrane protein-related signature yielded better predictive performances than traditional clinical variables

**Figure 5:**
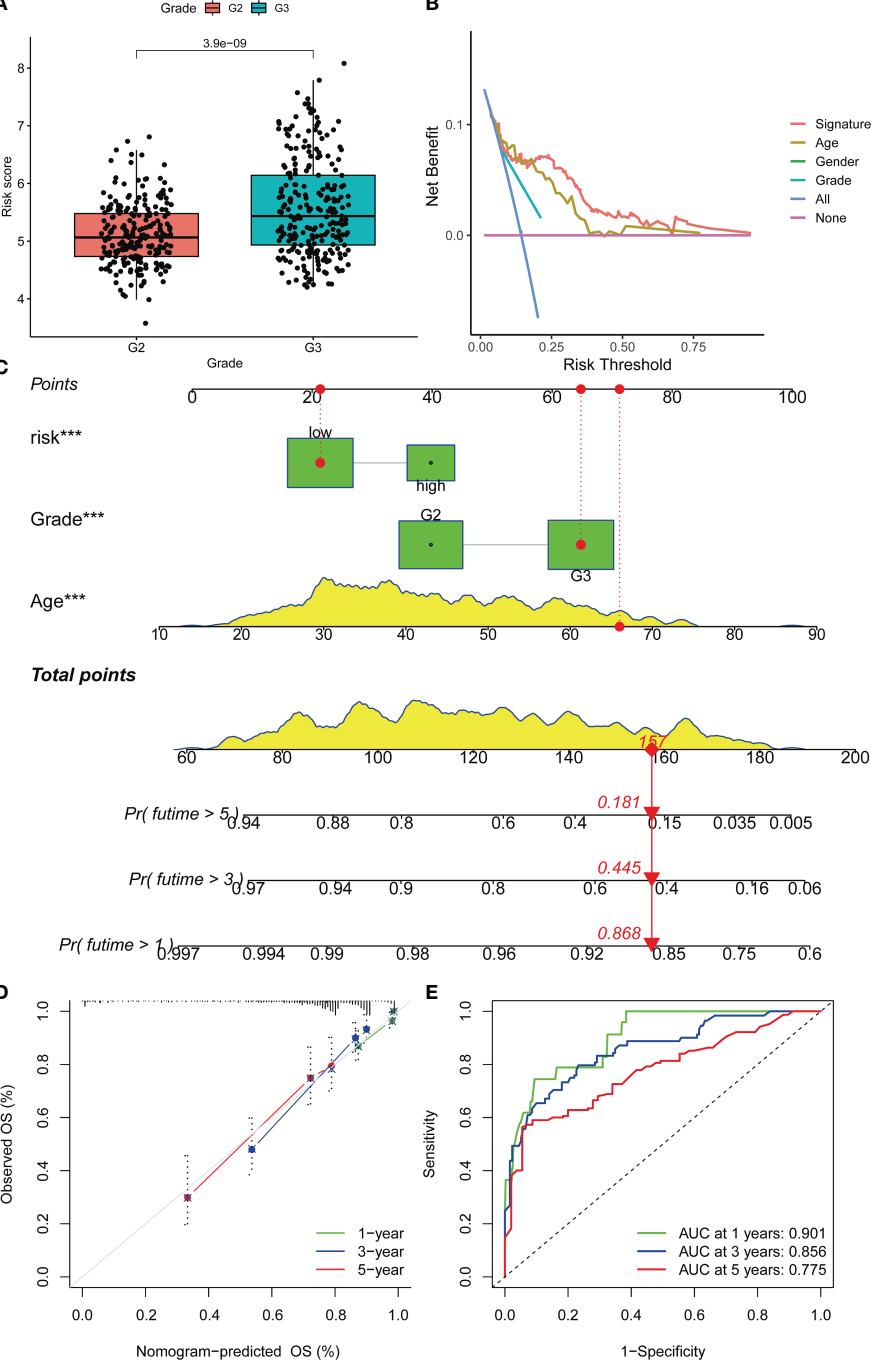
Prognostic prediction of glioma patients with other clinical factors based on the OS model. **(A)** Boxplots were used to display the difference in risk scores in different tumor grades. **(B)** Decision curve analysis of the constructed risk prediction model. Using the signature to predict patient survival can benefit the patient similar to other conventional clinical variables. **(C)** Nomogram integrating risk and clinical characteristics. **(D)** calibration of the nomogram at 1-year, 3- year and 5-year survival in the TCGA cohort. **(E)** Time-dependent ROC curve of the constructed nomogram model. Nomogram was built using the Multivariate proportional hazards model. Differential analyses were conducted using the Wilcoxon test. ***p < 0.001.

The staging index is an important clinicopathological feature of glioma. Our results revealed that the risk score had significant differences in G2 and G3 grades (p value< 0.001) (**Figure 5A**). DCA plots illustrated a net clinical benefit of the signature to predict glioma survival compared with traditional clinical variables (**Figure 5B**). Then, a nomogram was established to predict the OS for glioma patients (**Figure 5C**). Based on the results of multivariate analysis, a score was assigned for each predictor, and three factors were integrated into the nomogram to predict OS in patients with glioma. The calibration curve showed that the 1-, 2-, and 3-year OS predicted by the nomogram were in accordance with the actual outcomes (**Figure 5D**). Besides, the 1-, 2-, and 3-year AUC values were 0.901, 0.856 and 0.775, respectively (**Figure 5E**).

### Transmembrane protein-related genes may influence the development and prognosis of glioma by regulating the immune microenvironment

**Figure 6:**
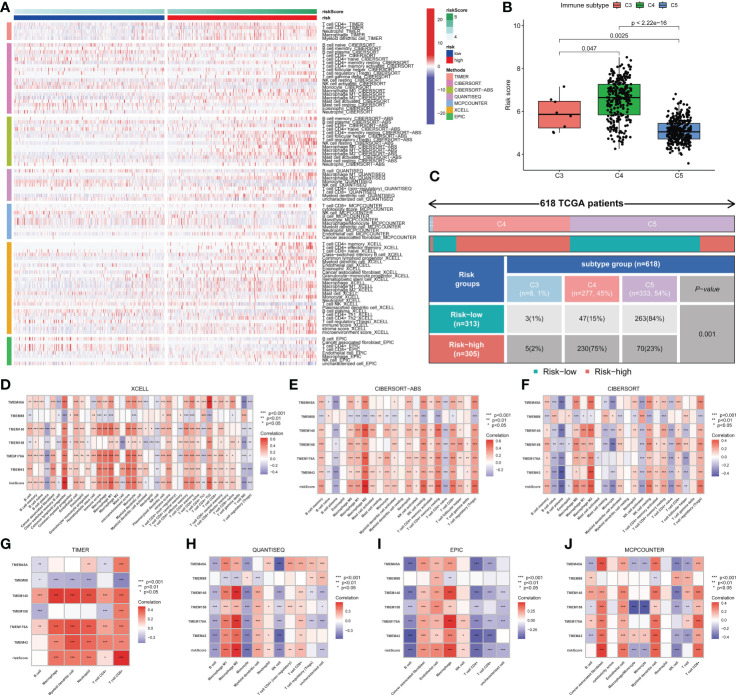
Immune cell infiltration in glioma. **(A)** Differences in immune cell infiltration between high- and low-risk patients. **(B)** Three immune types were identified in the TCGA-glioma dataset, namely Inflammatory (Immune C3), Lymphocyte Depleted (Immune C4) and Immunologically Quiet (Immune C5). Significant differences in risk scores were observed between different immune subtypes. **(C)** The association between risk groups with immune subtypes in the TCGA-Glioma cohort. **(D-J)** Correlation between risk score, genes in the model and different types of immune cell content based on 7 immune infiltration algorithms. Correlation analysis was conducted using Pearson correlation analysis. Differential analyses were conducted using the Wilcoxon test.

The high- and low-risk groups exhibited significant microenvironmental heterogeneity, and the high-risk group was associated with relatively more immune cell infiltration (**Figure 6A**). Glioma patients were identified as three immune subtypes, including inflammatory (immune C3), lymphocyte depleted (immune C4) and immunologically quiet (immune C5). C4 and C5 were the main types, while the latter had a higher risk score (**Figures 6B, C**). The risk score and six genes identified by the signature were correlated with the abundance of immune cells. Multi-software analysis showed that CD8+T cell, memory CD4+ T cell, Macrophage (M0, M1, M2), and Myeloid dendritic cell exhibited a low to moderate positive correlation with the risk score. Besides, a low to moderate negative correlation was found between B cells and the risk score (**Figures 6D–J**), demonstrating that transmembrane protein-related genes may influence the development and prognosis of glioma by regulating the immune microenvironment.

### TMEM158 was expressed in a variety of malignant cells

**Figure 7:**
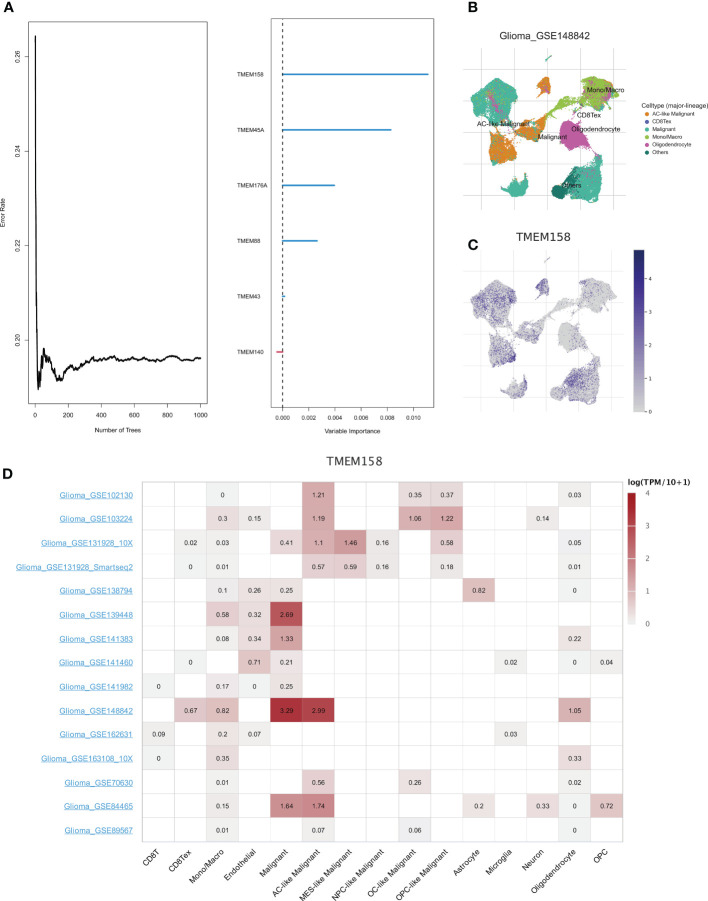
Identification of hub genes using the random forest algorithm. **(A)** The graph shows the error rate of the data as a function of the classification tree and the out-of-bag importance values for the predictors. **(B)** Annotation of all cell types in dataset GSE148842 based on the TISCH database. **(C, D)** Expressions of TMEM158 in GSE148842 and other Glioma single-cell datasets.

Further, we adopted the “randomForestSRC” R package to undergo feature selection. **Figure 7A** exhibited the relationship between the error rate and the number of classification trees and the out-of-bag feature importance of 6 genes, among which TMEM158 was the most important. The TISCH database was utilized to analyze the expression profile of TMEM158 based on the single-cell dataset Glioma_GSE148842 (**Figure 7B**). TMEM158 was mainly expressed in malignant cells but was low in oligodendrocytes (**Figure 7C**). Results from the 15 single-cell datasets were consistent, corroborating that TMEM158 was expressed in multiple malignant cells, especially in AC-like malignant cells (**Figure 7D**).

## Discussion

As the most prevalent primary tumor of the brain and spinal cord (27), glioma is widely thought to be derived from the neuroglial stem or progenitor cells (28). Due to the high recurrence rate and poor prognosis of glioma, it is essential to predict clinical outcomes to guide treatment strategies for this patient population. Conventional therapies, including surgery, chemotherapy, and radiotherapy, exhibit limitations in improving the clinical prognosis of glioma patients. The advent of immunotherapy has brought new hope, given its ability to penetrate the blood-brain barrier since the pioneering discovery of lymphatics in the central nervous system (29). However, not all patients can benefit from the long-term treatment course. Therefore, the quest for potent biomarkers should be emphasized for the optimal selection of patients for individualized treatment.

In this study, we found that the overall expression pattern of transmembrane protein-related genes was associated with malignant characteristics and the prognosis of glioma. Upon further validation, we inferred that these genes are significant for in-depth exploration of the pathogenesis of glioma. After the screening, six hub genes (TMEM45A, TMEM88, TMEM140, TMEM158, TMEM176A, and TMEM43) were identified, and a risk signature was constructed. The signature was validated using a series of approaches to more accurately and effectively predict clinical outcomes of glioma than traditional clinical variables and reflect clinicopathologic features associated with malignancy. In addition, we systematically analyzed the correlations between characteristic risk score-related genes and immune cell contents in gliomas and targeted the expression of hub genes based on 15 single-cell datasets. These findings may explain the poorer survival rates in the high-risk group to some extent and highlight the role of transmembrane protein-related genes in further stratifying the survival of glioma patients. Our transmembrane protein-related gene signature may be a vital auxiliary tool to assist clinicians in predicting the prognosis of glioma.

We analyzed the expression data of 14 transmembrane protein-related genes in glioma patients who were followed up for more than 30 days in the TCGA, CGGA and GEO databases and screened 9 transmembrane protein-related genes with prognostic significance. Finally, a 6-gene signature was built based on the risk genes TMEM45A, TMEM140, TMEM158, TMEM176A and TMEM43, and the protective gene TMEM88 by comparing their coefficient values. Over the years, these genes have been extensively studied, especially in some cancer types. In this respect, TMEM45A has been associated with various cancer features, such as cell proliferation, invasion, migration, and Epithelial-Mesenchymal Transition (EMT), and silencing TMEM45A can reverse cisplatin resistance (30–33). TMEM140 has been underexplored in the field of oncology. However, it can reportedly inhibit herpes simplex virus-1 (HSV-1) proliferation by selectively blocking the exit of the viral nucleocapsid during viral assembly (34). TMEM158 plays an important role in many cancers since it is upregulated in the renin-angiotensin system (Ras)-induced senescence process (35). Moreover, TMEM158 has been reported as the key regulator for tumorigenesis and drug resistance in colorectal cancer (36). What’s more, overexpression of TMEM158 is significantly associated with clinicopathologic features (including tumor size, TNM staging, and vascular infiltration) and poor prognosis of pancreatic cancer (PC) patients, and it could promote proliferation, migration, and invasion of PC cells through activation of transforming growth factor (TGF)-β1 and Phosphoinositide 3-Kinase (PI3K)/protein kinase B (AKT) signaling pathways (37).

Cuajungco MP et al. confirmed that human TMEM176A and 176B protein levels were significantly elevated in lymphoma and lung carcinoma but not in normal tissues, which substantiated that TMEM176A and 176B could be potential biomarkers for some human cancers (38). An increasing body of evidence suggests that TMEM176A can inhibit tumor cell growth and migration by constraining extracellular signal-regulated kinase (ERK) signal transduction in lung, pancreatic and liver cancer (39–41). Intriguingly, upregulated TMEM176A expression has been demonstrated to prevent dendritic cell (DC) maturation and inhibit DC activity in the general population when DCs have been shown to mediate recovery from central nervous system damage and/or protective autoimmunity (42). These findings might illustrate its potential role as a risk factor in glioma. Moreover, TMEM43 has been correlated with different diseases. For example, the deficiency of TMEM43 is widely thought to cause arrhythmogenic right ventricular cardiomyopathy type 5 (ARVD5) (43). Furthermore, Jiang C et al. verified that high TMEM43 expression was closely related to brain tumor malignancy, and inhibiting the expression of TMEM43 in brain tumor cells could lead to its growth *in vitro* and *in vivo* (8). In contrast, the TMEM88 gene was found to be a protective factor against glioma in the present study. Consistently, TMEM88 upregulation can result in the decreased ability of cell proliferation and invasion dramatically in bladder cancer, and nude mouse models substantiated that the overexpression of TMEM88 prevents tumor formation and growth of bladder cancer cells (44). Similar findings have been reported in thyroid cancer (45). Overall, patients in the low-risk group had higher OS than those in the high-risk group based our novel signature. External dataset and internal KM validations demonstrated that the signature had good predictive efficacy and was independent of other clinical traits. Importantly, our novel signature could enable clinicians to assess patient survival more accurately and effectively.

The tumor microenvironment is a complex system that plays an essential role in the proliferation and progression of tumor cells. Based on our signature, it was found that differential TME features were displayed in the high- and low-risk group. Moreover, the low-risk group was associated with more significant immune cell infiltration. Meanwhile, the low-risk group had a better prognosis. Additionally, some types of immune cells like CD8+T cells, CD4+ memory T cells, Macrophages (M0, M1, M2), and Myeloid DCs were positively correlated with risk scores. In contrast, B cells and risk scores were negatively correlated, possibly due to potential immune escape in the high-risk group. Glioma-associated microglia or macrophages and medullary suppressor cells were the most infiltrated cell types in the TME of gliomas, and their levels negatively correlated with the prognosis of cancer. In addition, bone marrow-derived suppressor cells inhibit NK cell-mediated cytotoxic responses (46). Immune cells perform immune surveillance functions through cell migration. It has been established that glioma has a rather complex tumor immune microenvironment (TIME). Interestingly, glioma-associated myeloid cells significantly promoted the invasiveness of glioma tumor cells (47). Moreover, a high infiltration of regulatory T cells is closely associated with poor clinical outcomes in glioma (48). A study revealed that in the TIME of glioma patients, immune cell infiltration was elevated in the high-risk group, with higher infiltration of tumor immune cells correlated with more advanced tumor grade (49), consistent with our findings.

However, the limitations of our study should be acknowledged. Our findings were based on retrospective data from TCGA, CGGA and GEO databases, with missing data on treatment and relapse. Indeed, *in vivo* or *in vitro* experiments and prospective clinical studies are warranted to validate our findings.

## Conclusions

In summary, this study identified a 6-gene signature with prognostic value for glioma patients. Our study presents a predictive model and biomarker for glioma patients and provides the foothold for further research to improve the outcomes of this patient population.

## Data availability statement

The original contributions presented in the study are included in the article/**Supplementary Material**. Further inquiries can be directed to the corresponding author.

## Author contributions

YZ and WZ carried out the analysis and made tables and figures. YZ, WZ, QY, WH, PY, TS, LF, JJ, and FS wrote and revised the manuscript. WC, YZ, and WZ conceived the study. All authors contributed to the article and approved the submitted version.

## Glossary

**Table:**

TCGA	The Cancer Genome Atlas
CGCA	Chinese Glioma Genome Atlas
GEO	Gene Expression Omnibus
ROC	receiver operating characteristic
C-index	Concordance-index
DCA	Decision Curve Analysis
PCA	Principal Component Analysis
OS	overall survival
TMEM	transmembrane protein
ER	endoplasmic reticulum
KM	Kaplan Meier
CDF	cumulative distribution function
MCP-Counter	Microenvironment Cell Populations-counter
ESTIMATE	Estimation of Stromal and Immune cells in Malignant Tumors using Expression data
GSVA	gene set variation analysis
TME	tumor microenvironment
TPRPGs	transmembrane proteins-related prognostic genes
TISCH	Tumor Immune Single-cell Hub
AUC	area under curve
EMT	Epithelial-Mesenchymal Transition
HSV-1	herpes simplex virus-1
RAS	renin-angiotensin system
PC	pancreatic cancer
TGF	transforming growth factor
PI3K	Phosphoinositide 3-Kinase
AKT	protein kinase B
ERK	extracellular signal-regulated kinase
DC	dendritic cell
ARVD5	arrhythmogenic right ventricular cardiomyopathy type 5
TIME	tumor immune microenvironment
